# Bio-based production of organic acids with *Corynebacterium glutamicum*

**DOI:** 10.1111/1751-7915.12013

**Published:** 2012-12-02

**Authors:** Stefan Wieschalka, Bastian Blombach, Michael Bott, Bernhard J Eikmanns

**Affiliations:** 1 Institute of Microbiology and Biotechnology, University of UlmD-89069, Ulm, Germany; 2 Institute of Biochemical Engineering, University of StuttgartD-70569, Stuttgart, Germany; 3 Institute for Bio-und Geosciences, IBG-1: Biotechnology, Forschungszentrum JülichD-52425, Jülich, Germany

## Abstract

The shortage of oil resources, the steadily rising oil prices and the impact of its use on the environment evokes an increasing political, industrial and technical interest for development of safe and efficient processes for the production of chemicals from renewable biomass. Thus, microbial fermentation of renewable feedstocks found its way in white biotechnology, complementing more and more traditional crude oil-based chemical processes. Rational strain design of appropriate microorganisms has become possible due to steadily increasing knowledge on metabolism and pathway regulation of industrially relevant organisms and, aside from process engineering and optimization, has an outstanding impact on improving the performance of such hosts. *Corynebacterium glutamicum* is well known as workhorse for the industrial production of numerous amino acids. However, recent studies also explored the usefulness of this organism for the production of several organic acids and great efforts have been made for improvement of the performance. This review summarizes the current knowledge and recent achievements on metabolic engineering approaches to tailor *C. glutamicum* for the bio-based production of organic acids. We focus here on the fermentative production of pyruvate, l-and d-lactate, 2-ketoisovalerate, 2-ketoglutarate, and succinate. These organic acids represent a class of compounds with manifold application ranges, e.g. in pharmaceutical and cosmetics industry, as food additives, and economically very interesting, as precursors for a variety of bulk chemicals and commercially important polymers.

**Funding Information** Work in the laboratories of the authors was supported by the Fachagentur Nachwachsende Rohstoffe (FNR) of the Bundesministerium für Ernährung, Landwirtschaft und Verbraucherschutz (BMELV; FNR Grants 220-095-08A and 220-095-08D; Bio-ProChemBB project, ERA-IB programme), by the Deutsche Bundesstiftung Umwelt (DBU Grant AZ13040/05) and the Evonik Degussa AG.

## Introduction

The depletion of earth's fossil energy resources, accompanied by the strong impact of their use on the environment, particularly in form of higher CO_2_ emissions, raises the demand and the consumer pull for sustainable, safe and efficient substitution of hitherto crude oil derived chemicals and chemical building blocks from renewable resources. Besides chemical manufacturing of renewable feedstocks to valuable compounds, biotechnological processes afford more and more opportunities to produce fuels, building blocks, and solvents in a cost-effective way from biomass (Bozell and Petersen, 2010). Chemical buildings blocks, such as some organic acids, serve as precursors for a variety of bulk chemicals and commercially important polymers (Werpy and Petersen, 2004). The cost-effective bio-based production of these chemicals is a most relevant goal for the future and has to meet economic and environmental requirements. Therefore, the microbial production systems have to perform excellent with regard to yield, productivity, product purity and flexibility to substrate consumption.

*Corynebacterium glutamicum* is a Gram-positive facultative anaerobic organism that grows on a variety of sugars, organic acids, and alcohols as single or combined carbon and energy sources (Eggeling and Bott, 2005; Liebl, 2006; Nishimura *et al*., 2007; Takeno *et al*., 2007). The organism is generally regarded as safe (GRAS status) and is traditionally employed for large scale production of amino acids, such as l-glutamate (> 2 million t/a) and l-lysine (> 1.4 million t/a) (Eggeling and Bott, 2005; Takors *et al*., 2007; Ajinomoto, 2010; 2011). In order to improve the production performance by metabolic engineering approaches, the central carbon metabolism, the physiology and the regulation of main and specific pathways of *C. glutamicum* were analysed in detail and genetic tools as well as systems biology approaches on the ‘omics’ level have been developed and employed (overviews in Kirchner and Tauch, 2003; Eggeling and Bott, 2005; Sauer and Eikmanns, 2005; Wendisch *et al*., 2006a; Bott, 2007; Takors *et al*., 2007; Burkowski, 2008; Brinkrolf *et al*., 2010; Becker and Wittmann, 2011; Teramoto *et al*., 2011; Vertes *et al*., 2012). Since *C. glutamicum* is regarded as a robust and easily manageable production host, recent studies also focused on the suitability of this organism for the production of other commodity chemicals, such as the biofuels isobutanol and ethanol (Inui *et al*., 2004b; Smith *et al*., 2010; Blombach and Eikmanns, 2011; Blombach *et al*., 2011), the diamines cadaverine and putrescine (Mimitsuka *et al*., 2007; Schneider and Wendisch, 2010; 2011; Kind *et al*., 2010a,2010b; Kind and Wittmann, 2011), the sugar alcohol xylitol (Sasaki *et al*., 2010), gamma-amino butyric acid (Takahashi *et al*., 2012), polyhodroxybutyrate (Song *et al*., 2012), and also several organic acids (reviewed in this article).

**Figure 1:**
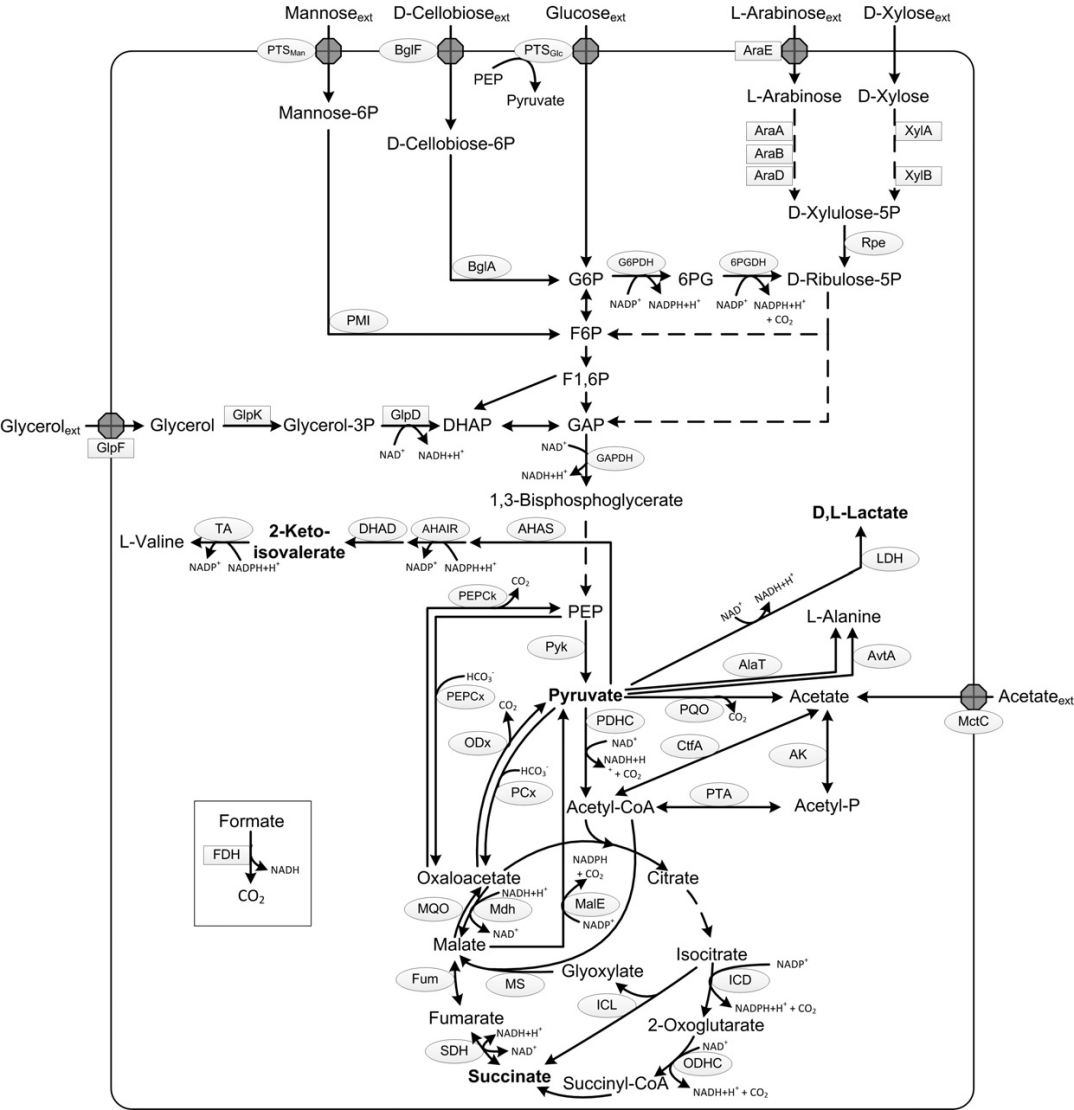
Schematic presentation of the central carbon metabolism of *C. glutamicum* including pathways for the degradation of carbon sources (glucose, glycerol, d-cellobiose, l-arabinose, d-xylose, mannose, formate, acetate) used for the production of pyruvate, d,l-lactate, 2-ketoisovalerate, 2-ketoglutarate and succinate. Ellipses represent enzymes and transport systems present in *C. glutamicum*. Rectangles represent heterologous enzymes. *Abbreviations*: Coding genes are given in brackets. 6PG, 6P-gluconate; 6PGDH (*gnd*), 6PG dehydrogenase; AHAIR (*ilvC*), acetohydroxyacid isomeroreductase; AHAS (*ilvBN*), acetohydroxyacid synthase; AK (*ack*), acetate kinase; AlaT (*alaT*), alanine aminotransferase; AraA (*araA* from *E. coli*), arabinose isomerase; AraB (*araB* from *E. coli*), ribulokinase; AraD (*araD* from *E. coli*), l-ribulose-5-phosphate 4-epimerase; AraE (*araE* from *E. coli*), l-arabinose transporter; AvtA (*avtA*), valine-pyruvate aminotransferase; BglA (*bglA1*, *bglA2*), phospho-β-glucosidases; BglF (*bglF*^V317A^), mutated PTS permease enabling d-cellobiose import; CtfA (*cat*), CoA transferase A; DHAD (*ilvD*), dihydroxyacid dehydratase; DHAP, dihydroxyacetone-P; F1,6P, fructose-1,6P; F6P, fructose-6P; FDH (*fdh* from *Mycobacterium vaccae*), formate dehydrogenase; Fum (*fum*), fumarase; GAP, glyceraldehyde-3P; GAPDH (*gapA*), GAP dehydrogenase; GlpD (*glpD* from *E. coli*), glycerol-3P dehydrogenase; GlpF (*glpF* from *E. coli*), glycerol facilitator; GlpK (*glpK* from *E. coli*), glycerol kinase; G6P, glucose-6P; G6PDH (*zwf*, *opcA*), G6P dehydrogenase; ICD (*icd*), isocitrate dehydrogenase; ICL (*aceA*), isocitrate lyase; LDH (native *ldhA* or *ldhA* from *L. delbrueckii*), l-and d-lactate dehydrogenase respectively; MalE (*malE*), malic enzyme; MctC (*mctC*) monocarboxylic acid transporter; Mdh (*mdh*), malate dehydrogenase; MQO (*mqo*), malate:quinone oxidoreductase; MS (*aceB*), malate synthase; ODHC (*odhA*, *aceF*, *lpd*), 2-oxoglutarate dehydrogenase complex; ODx (*odx*), oxaloacetate decarboxylase; PCx (*pyc*), pyruvate carboxylase; P, phosphate; PDHC (*aceE*, *aceF*, *lpd*), pyruvate dehydrogenase complex; PEP phosphoenolpyruvate; PEPCk (*pck*), PEP carboxykinase; PEPCx (*ppc*), PEP carboxylase; Pyk (*pyk*), pyruvate kinase; PMI (*manA*), phosphomannose isomerase; PQO (*pqo*), pyruvate: quinone oxidoreductase; PTA (*pta*), phosphotransacetylase; PTS (*ptsG*, *hpr*; *ptsI*), phosphotransferase system; Rpe (*rpe*), ribulose-5-phosphate epimerase; SDH (*sdhABC*), succinate dehydrogenase; TA (*ilvE*), transaminase B; XylA (*xylA* from *E. coli*), xylose isomerase; XylB (*xylB* from *E. coli*), xylulokinase.

Six years ago, Wendisch and colleagues (2006b) reviewed the metabolic engineering of *C. glutamicum* and *Escherichia coli* for the biotechnological production of organic acids and amino acids. At that time, *E. coli* was the superior platform organism for the production of organic acids and it was hardly known that *C. glutamicum* forms lactate and succinate under oxygen-deprivation conditions (Dominguez *et al*., 1993; Inui *et al*., 2004a; Okino *et al*., 2005). However, it was foreseeable that genetically modified *C. glutamicum* strains will become promising biocatalysts for the production of at least some organic acids. As outlined in the following, great efforts have been made in the last 7 years to successfully implement and/or to improve the production of several organic acids with *C. glutamicum*. This review focuses on the metabolic/genetic engineering approaches to tailor *C. glutamicum* for the fermentative production of pyruvate, d-and l-lactate, 2-ketoisovalerate, 2-ketoglutarate, and succinate from renewable carbon sources. Figure 1 gives an overview on pathways and enzymes of the central metabolism of *C. glutamicum*, including the pathways for the degradation of selected substrates and those for the synthesis of organic acids produced with this organism.

## Production of pyruvate

Pyruvate is broadly used as ingredient or additive in food, cosmetics and pharmaceuticals, but also for the synthesis of various chemicals and polymers (Li *et al*., 2001; Zhu *et al*., 2008). Chemical production of pyruvate is realized by dehydration and decarboxylation of tartaric acid, but in a cost-ineffective way (Howard and Fraser, 1932; Li *et al*., 2001). Different approaches were made for pyruvate production with eukaryotic microorganisms like multi-auxotrophic yeasts (reviewed in Li *et al*., 2001); however, prokaryotic microorganisms, such as *E. coli* and *C. glutamicum*, also were successfully engineered to produce pyruvate.

**Figure 2:**
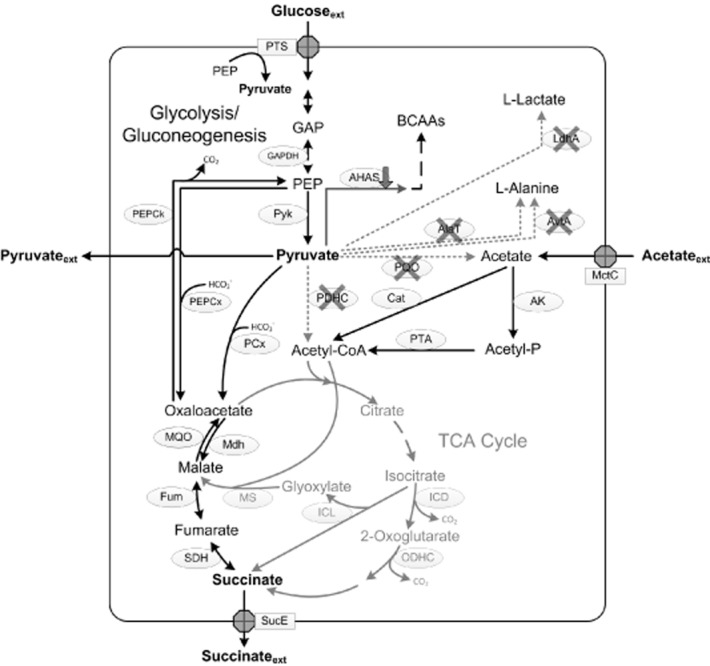
Schematic presentation of the central carbon metabolism of *C. glutamicum* ELB-P with the corresponding enzymes and modifications, leading to pyruvate production under aerobic conditions and reductive succinate production under anaerobic conditions. For most abbreviations see legend to Fig. 1. Further abbreviations: BCAAs, branched-chain amino acids; LdhA, NAD^+^-dependent l-lactate dehydrogenase; SucE, succinate exporter; TCA, tricarboxylic acid. Down arrow at AHAS indicates decreased activity of the truncated AHAS derivative, crosses indicate inactivation of the enzyme by deletion of the respective gene. Dotted arrows indicate pathways not present due to gene inactivation, grey arrows indicate pathways not used in *C. glutamicum* ELB-P.

Pyruvate is a central intermediate in the carbon and energy metabolism (see Fig. 1) in all organisms and thus, for construction of an efficient pyruvate-producing *C. glutamicum* strain, the major pyruvate-drawing reactions had to be downregulated or even eliminated. In the course of the molecular analysis of the pyruvate dehydrogenase complex (PDHC), Schreiner and colleagues (2005) inactivated this complex in *C. glutamicum* by deletion of the *aceE* gene, encoding the E1p subunit of the PDHC. The resulting strain *C. glutamicum* Δ*aceE* required acetate or ethanol as an additional carbon source for growth on glucose (Schreiner *et al*., 2005; Blombach *et al*., 2009). In an approach to engineer *C. glutamicum* for l-valine production, Blombach and colleagues (2007) observed that *C. glutamicum* Δ*aceE* showed a relatively high intracellular concentration of pyruvate and, when acetate was exhausted from the medium and growth stopped, secreted significant amounts of l-alanine (30 mM), l-valine (30 mM), and pyruvate (30 mM) from glucose. In subsequent studies, the PDHC-deficient strain turned out to be an excellent starting point to engineer *C. glutamicum* for the efficient production of l-valine (Blombach *et al*., 2007; 2008; 2009; Krause *et al*., 2009), isobutanol (Blombach *et al*., 2011), and also of 2-ketoisovalerate (Krause *et al*., 2010; see below), succinate (see below) and pyruvate. The additional inactivation of the pyruvate:quinone oxidoreductase (PQO) and NADH-dependent l-lactate dehydrogenase (l-LDH) significantly improved pyruvate formation (Wieschalka *et al*., 2012). In shake-flask experiments, *C. glutamicum* Δ*aceE* Δ*pqo* Δ*ldhA* accumulated in a growth-decoupled manner about 50 mM pyruvate with a substrate-specific product yield (Y_P/S_) of 0.48 mol per mol of glucose, aside from l-alanine (29 mM) and l-valine (21 mM) as by-products (Wieschalka *et al*., 2012). To abolish overflow metabolism towards l-valine, the native acetohydroxyacid synthase (AHAS) was substituted by a leaky variant (ΔC-T IlvN) leading to an almost threefold increased Y_P/S_ of 1.36 mol pyruvate per mol of glucose, and a strong increase of pyruvate production (up to 193 mM), while l-valine and l-alanine formation were reduced to 1 mM and 9 mM respectively (Wieschalka *et al*., 2012). Additional deletion of the genes encoding alanine aminotransferase (AlaT) and valine-pyruvate aminotransferase (AvtA) resulted in cumulative reduction of l-alanine as undesired by-product by 50% (Wieschalka *et al*., 2012). With the final strain *C. glutamicum* Δ*aceE* Δ*pqo* Δ*ldhA* ΔC-T *ilvN* Δ*alaT* Δ*avtA* (designated as *C. glutamicum* ELB-P; see Fig. 2) up to 200 mM pyruvate were formed in shake-flask experiments, with a Y_P/S_ of 1.49 mol per mol of glucose. The yields of the by-products l-alanine and l-valine were evanescent low with 0.03 and 0.01 mol per mol of glucose respectively (Wieschalka *et al*., 2012). To study the relevance for industrial applications, fed-batch fermentations were performed with *C. glutamicum* ELB-P. When *C. glutamicum* ELB-P was cultivated with a constant pO_2_ of about 30% a twofold lower glucose consumption rate (0.28 mmol g cell dry weight _(CDW)_^−1^ h^−1^) and a significantly lower Y_P/S_ (0.8 mol pyruvate per mol of glucose) were observed when compared with shake-flask experiments (0.58 mmol g_(CDW)_^−1^ h^−1^ and 1.49 mol pyruvate per mol of glucose respectively). Implementation of low oxygen tension from the middle until the end of growth phase restored the production performance and led to the formation of more than 500 mM (45 g l^−1^) pyruvate with a Y_P/S_ of 0.97 mol pyruvate per mol of glucose in the production phase (Wieschalka *et al*., 2012). In comparison, the best pyruvate-producing *E. coli* strains (*E. coli* YYC202 and ALS1059) produced under optimized process conditions about 720 mM (63 g l^−1^) and 1 M (90 g l^−1^) pyruvate, with Y_P/S_ of 1.74 and 1.39 mol pyruvate per mol of glucose respectively (Zelic *et al*., 2003; Zhu *et al*., 2008). Since the yield of *C. glutamicum* ELB-P in shake-flask experiments is in the same range as in these *E. coli* strains, further process optimization might disclose the whole potential of *C. glutamicum* ELB-P for a further improved pyruvate production process.

## Production of lactate

Lactate is widely used as both d-and l-isomers for pharmaceutical, cosmetic, leather and textile, chemical, biomedical and food industries, as well as for green solvent and biodegradable fibre and polymer production (Hofvendahl and Hahn-Hägerdal, 2000; Bozell and Petersen, 2010; Okano *et al*., 2010). Especially the latter, in form of the stereocomplex of d-and l-polylactic acid is a fully biodegradable substitute for polyethylene terephthalates with high melting temperatures and mechanical strength, and therefore, of great economical interest (Lorenz and Zinke, 2005; Dodds and Gross, 2007; Fukushima *et al*., 2007; Uehara *et al*., 2010). In the past and still today, wild-type and recombinant lactic acid bacteria have been mainly employed for the production of both d-and l-lactate (reviewed in Okano *et al*., 2010). However, these bacteria have a demand for complex media, which makes the cultivation of the organisms and the purification of the product relatively cost-intensive. Therefore, other less fastidious organisms, such as metabolically engineered *E. coli*, *Saccharomyces cerevisiae* and *C. glutamicum* have also been developed for efficient l-and d-lactic acid production (Okano *et al*., 2010).

*Corynebacterium glutamicum* is facultatively anaerobic and grows aerobically and anaerobically in the presence of oxygen and nitrate respectively (Nishimura *et al*., 2007; Takeno *et al*., 2007). The lack of oxygen or nitrate as external electron acceptors results in growth arrested cells, which still have the capability to ferment C6 sugars to l-lactate and succinate as major products. Dominguez and colleagues (1993) firstly reported that *C. glutamicum* forms lactate, succinate and acetate at small amounts when oxygen is limited during aerobic growth. Inui and colleagues (2004a) further studied this phenomenon in an attempt to utilize corynebacterial properties for the industrial production of lactate and succinate. These authors reported of organic acid production with *C. glutamicum* strain R and described that the bacteria showed no growth under oxygen-deprivation conditions, but produced significant amounts of l-lactate (∼ 220 mM) and succinate (∼ 20 mM) from about 130 mM glucose. Addition of bicarbonate to the medium led to an increase of the NAD^+^/NADH ratio and, probably as consequence of a derepression of the glyceraldehyde-3-phosphate dehydrogenase gene *gapA*, to an increased glucose consumption (Inui *et al*., 2004a). Furthermore, the addition of bicarbonate led to an altered product spectrum, i.e. the formation of succinate and lactate increased by a factor of two to four and significant concentrations (about 10 mM) of acetate were formed (Inui *et al*., 2004a; Okino *et al*., 2005). In a high cell density [30 g _dry cell weight (DCW)_ l^−1^] fed-batch system, *C. glutamicum* R already produced 574 mM l-lactate (i.e. 53 g l^−1^), with only small amounts (< 10 mM) of succinate and acetate as side-products (Okino *et al*., 2005). Addition of 400 mM bicarbonate raised the l-lactate concentration to more than 1 M (97.5 g l^−1^), but also the concentrations of the by-products succinate (192 mM) and acetate (50 mM) (Okino *et al*., 2005). Even without genetic modification of *C. glutamicum*, the resulting l-lactate titre from glucose and the Y_P/S_ of 1.79 mol l-lactate per mol of glucose (i.e. 0.90 g g^−1^) are highly competitive as e.g. the best known metabolically engineered l-lactate-producing *E. coli* strain SZ85 (*pflB*, *frdBC*, *adhE*, *ackA*, *ldhA::ldhL*, overexpressed *ldhL* gene from *Pediococcus acidilactici*) accumulated 505 mM l-lactate (i.e. 46 g l^−1^) with an optical purity of > 99% and a Y_P/S_ of 1.9 mol per mol of glucose (i.e. 0.95 g g^−1^; Zhou *et al*., 2003b) (see Table 1).

**Table 1:** Maximal titres, substrate-specific yields (Y_P/S_), productivities, by-products and the respective references of the so far most efficient processes for organic acid production with *C. glutamicum* and *E. coli* strains

Strain	Medium	Maximal titre (mM) (g l^−1^)	Y_P/S_ (mol_product_ per mol_substrate_) (g g^−1^)	Productivity^a^ (mM h^−1)^ (g l^−1^ h^−1^)	By-products^b^	Reference
Pyruvate						
*C. glutamicum* ELB-P	minimal medium, **glucose**	512 (44.5)	1.49 (0.72)	5.6 (0.49)	–c	Wieschalka *et al*. (2012)
*E. coli* ALS1059	minimal medium, **glucose**, l-isoleucine, betaine	1022 (88.9)	1.39 (0.67)	23.9 (2.08)	–	Zhu *et al*. (2008)
*E. coli* YYC202	minimal medium, **glucose**	720 (62.6)	1.74 (0.84)	37.0 (3.22)	–	Zelic *et al*. (2003)
l-Lactate						
*C. glutamicum* R	minimal medium, **glucose**	574 (51.1)	1.42 (0.70)	71.8 (6.39)	–	Okino *et al*. (2005)
*C. glutamicum* R	minimal medium, **glucose**, bicarbonate	1061 (94.4)	1.79 (0.89)	176.8 (15.74)	acetate, succinate	Okino *et al*. (2005)
*E. coli* SZ85	minimal medium, **glucose**	505 (44.9)	1.90 (0.94)	7.2 (0.64)	–	Zhou *et al*. (2003b)
d-Lactate						
*C. glutamicum* R Δ*ldhA* pCRB204	minimal medium, **glucose**	1340 (119.3)	1.73 (0.86)	44.5 (3.96)	acetate, succinate	Okino *et al*. (2008b)
*E. coli* JP203	complex medium, **glucose**	691 (61.5)	1.80 (0.89)	11.6 (1.03)	–	Chang *et al*. (1999)
*E. coli SZ63*	minimal medium, **glucose**	528 (47.0)	1.92 (0.95)	9.8 (0.87)	–	Zhou *et al*. (2003a)
2-Ketoisovalerate						
*C. glutamicum* Δ*aceE* Δ*pqo* Δ*ilvE* (pJC4ilvBNCD)	minimal medium, **glucose**, yeast extract	188 (21.8)	0.56 (0.36)	4.6 (0.53)	l-valine	Krause *et al*. (2010)
2-Ketoglutarate						
*C. glutamicum* R Δ*gdh* Δ*gltB* Δ*aceA*	complex medium, glucose, molasses, soybean hydrolysate	325 (47.5)	n.s.^d^	2.7 (0.39)	–	Jo *et al*. (2012)
Succinic acid (anaerobic)						
*C. glutamicum* R Δ*ldhA* pCRA717	minimal medium, **glucose**, bicarbonate	1240 (146.3)	1.40 (0.92)	27 (3.19)	acetate	Okino *et al*. (2008a)
*C. glutamicum* ELB-P	minimal medium, **glucose**	330 (38.9)	1.02 (0.67)	5.6 (0.66)	pyruvate	S. Wieschalka and B.J. Eikmanns, own unpubl. data
*C. glutamicum* BOL-3/pAN6-*gap*	saline, **glucose**, **formate**, bicarbonate	1134 (133.8)	1.67 (1.09)	21 (2.48)	2-oxoglutarate, acetate, fumarate, malate	Litsanov *et al*. (2012b)
*E. coli* SBS550MG/pHL413	complex medium, **glucose**	330 (38.9)	1.61 (1.06)	10 (1.18)	acetate, formate	Sánchez *et al*. (2005)
*E. coli* KJ134	minimal medium, **glucose**	606 (71.5)	1.53 (1.00)	6.4 (0.76)	acetate, pyruvate, malate	Jantama *et al*. (2008)
Succinic acid (aerobic)						
*C. glutamicum* BL-1/pAN6-*pyc*^P458S^*ppc*	minimal medium, **glucose**	90 (10.6)	0.45 (0.30)	0.8 (0.09)	2-oxoglutarate, acetate, pyruvate	Litsanov *et al*. (2012a)
*C. glutamicum* BL-1 pVWEx1-*glpFKD*	minimal medium, **glycerol**	79 (9.3)	0.21 (0.27)	3.6 (0.42)	acetate	Litsanov *et al*. (2012c)
*E. coli* HL51276k(pKK313)	complex medium, **glucose**, bicarbonate	70 (8.3)	1.09 (0.71)	1.2 (0.14)	acetate, pyruvate	Lin *et al*. (2005)
*E. coli* HL27659k(pKK313)	complex medium, **glucose**, bicarbonate	60 (7.1)	0.95 (0.62)	2.3 (0.27)	acetate	Lin *et al*. (2005)

a During production phase.

b Significant concentrations above 10 mM.

c – = byproducts below 10 mM.

d n.s. = not specified.

For d-lactate production with *C. glutamicum*, a l-LDH-deficient mutant was constructed, expressing the d-lactate dehydrogenase (d-LDH) from *Lactobacillus delbrueckii* (Okino *et al*., 2008b). Under oxygen-deprivation conditions, this mutant (*C. glutamicum* R Δ*ldhA*/pCRB204) produced in a high cell density system (60 g_(DCW)_ l^−1^) about 1.34 M d-lactate (i.e. 120 g l^−1^) within 30 h with an optical purity of > 99.9% and a Y_P/S_ of 1.73 mol per mol of glucose. But also significant amounts of succinate (146 mM) and actetate (52 mM) were formed, underlining product purity as major problem (Okino *et al*., 2008b). However, *C. glutamicum* R Δ*ldhA*/pCRB204 produced more d-lactate than *E. coli* JP203 (*pta*, *ppc*) (Chang *et al*., 1999) and SZ63 (W3110; *pflB*, *frdBC*, *adhE*, *ackA*) (Zhou *et al*., 2003a) (Table 1), the best known genetically defined d-lactate producing *E. coli* strains, harbouring the native d-LDH of *E. coli*. With about 690 mM (62 g l^−1^) and 530 mM (48 g l^−1^), these strains formed approximately half of the titre obtained with *C. glutamicum* R Δ*ldhA*/pCRB204, however, with comparable Y_P/S_ of between 1.76 and 1.92 mol d-lactate per mol of glucose (0.90–0.99 g d-lactate per g of glucose; Chang *et al*., 1999; Zhou *et al*., 2003a).

It has to be noted that all described *C. glutamicum* and *E. coli* processes have to compete with those using recombinant yeast strains (*Saccharomyces* and *Kluyveromyces*) that produce l-lactic acid with titres of up to 1.3 M, optical purity of > 99.9%, and Y_P/S_ of up to 1.6 mol l-lactate per mol of glucose (Saitoh *et al*., 2005; Okano *et al*., 2010).

## Production of 2-ketoisovalerate and 2-ketoglutarate

In nature, 2-ketoisovalerate (3-methyl-2-oxobutanoic acid) is a precursor for l-valine, l-leucine, and pantothenate synthesis in bacteria and plants. In these organisms, it is formed from two molecules of pyruvate via the reactions catalysed by AHAS, acetohydroxyacid isomeroreductase (AHAIR), and dihydroxyacid dehydratase (DHAD) (see Fig. 1). 2-Ketoisovalerate is used as substitute for l-valine or l-leucine in chronic kidney disease patients (Teschan *et al*., 1998; Feiten *et al*., 2005; Aparicio *et al*., 2009; 2012) and also has been used in therapy for uremic hyperphosphatemia (Schaefer *et al*., 1994). To our knowledge, 2-ketoisovalerate for these purposes is mainly synthesized chemically by different methods (Cooper *et al*., 1983) and only very recently, directed fermentative production of 2-ketoisovalerate with microorganisms has been reported for the first time (Krause *et al*., 2010; see below).

Since 2-ketoisovalerate stems from two molecules of pyruvate (see Fig. 1) and a PDHC-deficient *C. glutamicum* secreted significant amounts of pyruvate and l-valine (see above), *C. glutamicum* Δ*aceE* was an excellent basis to engineer *C. glutamicum* for the production of this 2-ketoacid. To avoid transamination of 2-ketoisovalerate to l-valine, the *ilvE* gene encoding transaminase B was deleted, leading to an auxotrophy for branched chain amino acids. Aerobically, *C. glutamicum* Δ*aceE* Δ*ilvE* formed about 76 mM pyruvate, 25 mM l-alanine, and 40 mM 2-ketoisovalerate in a growth-decoupled manner from glucose (Krause *et al*., 2010). Overexpression of the AHAS, AHAIR and DHAD genes shifted the product spectrum towards 2-ketoisovalerate and the resulting strain *C. glutamicum* Δ*aceE* Δ*ilvE* (pJC4ilvBNCD) produced in fed-batch fermentations about 85 mM 2-ketoisovalerate with a volumetric productivity of 1.9 mM h^−1^ and a Y_P/S_ of about 0.38 mol per mol of glucose. Although the PQO has been found to be dispensable for growth and a deletion was only slightly beneficial on l-valine production (Schreiner *et al*., 2006; Blombach *et al*., 2008), PQO inactivation turned out to be highly beneficial for 2-ketoisovalerate production. Compared with the parental strain, *C. glutamicum* Δ*aceE* Δ*ilvE* Δ*pqo* (pJC4ilvBNCD) showed in fed-batch fermentations more than two times higher final titres (up to 220 mM = 25.5 g l^−1^) and volumetric productivities of 4.6 mM h^−1^ (Krause *et al*., 2010; Table 1).

It is noteworthy to mention that the 2-ketoisovalerate-producer *C. glutamicum* Δ*aceE* Δ*ilvE* Δ*pqo* (pJC4ilvBNCD) was used as a basis for the generation of a series of *C. glutamicum* strains producing isobutanol via the so-called ‘Ehrlich pathway’ (Blombach and Eikmanns, 2011; Blombach *et al*., 2011). The most promising strain of this series, *C. glutamicum* Iso7, carries additional deletions of the l-LDH and malate dehydrogenase genes (Δ*ldhA* and Δ*mdh* respectively) and overexpresses additionally the *E. coli* transhydrogenase genes *pntAB*, the *Lactococcus lactis* ketoacid decarboxylase gene *kivD*, and the homologous alcohol dehydrogenase gene *adhA* (Blombach *et al*., 2011).

2-Ketoglutarate is an intermediate of the tricarboxylic acid (TCA) cycle and the precursor for the synthesis of glutamate and the glutamate family of amino acids. 2-Ketoglutarate is used in dairy industry (Banks *et al*., 2001; Gutiérrez-Mendéz *et al*., 2008) and also is suitable to treat chronic renal insufficiency in hemodialysis patients (Riedel *et al*., 1996). An enzymatic process to synthesize 2-ketoglutarate from glutamate via the coupled reactions of glutamate dehydrogenase and NADH oxidase has been established (Ödmann *et al*., 2004), however, this bioconversion seems not very efficient. Therefore, Jo and colleagues (2012) very recently used a glutamate-overproducing mutant of *C. glutamicum* for the construction of a 2-ketoglutarate-producer. Inactivation of the genes encoding glutamate dehydrogenase, glutamate synthase and isocitrate lyase (*gdh*, *gltB*, and *aceA* respectively) led to a drastic reduction of glutamate formation (< 10 mM) and concomitantly to 2-ketoglutarate accumulation to concentrations of up to 325 mM (47.5 g l^−1^) after 120 h of cultivation in medium containing glucose, molasses, glutamate, and soybean hydrolysate (Jo *et al*., 2012). To our knowledge, there were no other approaches to produce 2-ketoglutarate by fermentation with any other bacterium. However, Zhou and colleagues (2012) recently reported efficient 2-ketoglutarate production (up to about 380 mM) with a recombinant (‘non-conventional’) yeast strain of *Yarrowia lipolytica* with enhanced acetyl-CoA availability.

## Production of succinate

The C4 dicarboxylate succinate has been denoted as ‘a LEGO® of chemical industry’ (Sauer *et al*., 2008) and as such, can be used as precursor for known petrochemical bulk products, such as 1,4-butanediol, tetrahydrofuran, γ-butyrolactone, adipic acid, maleic anhydride, various n-pyrrolidinones, and linear aliphatic esters (Zeikus *et al*., 1999; Sauer *et al*., 2008; Bozell and Petersen, 2010). Moreover, succinate (or succinic acid) is directly used as surfactant, ion chelator, and as an additive in pharmaceutical, and food industry (McKinlay *et al*., 2007). The market potential for succinic acid and its direct derivatives has been estimated to be 245 000 tons per year, that for succinic acid-derived polymers about 25 000 000 tons per year, and with the transition to cost-efficient bio-based production of succinate or succinic acid, the market is predicted to steadily increase (Werpy and Petersen, 2004; Bozell and Petersen, 2010).

Aside from l-lactate and acetate, succinate is a natural fermentative end-product of the wild type of *C. glutamicum*, when incubated with glucose under oxygen deprivation (Dominguez *et al*., 1993; Inui *et al*., 2004a). Under these conditions, succinate is formed via glycolysis, carboxylation of phosphoenolpyruvate (PEP) or pyruvate to oxaloacetate (OAA) by PEP carboxylase (PEPCx) and/or pyruvate carboxylase (PCx), and subsequent conversion of OAA by malate dehydrogenase (Mdh), fumarase (Fum), and succinate dehydrogenase (SDH) (Inui *et al*., 2004a; see Fig. 1).

A two-stage succinate production process with *C. glutamicum* strain R was developed by Okino and colleagues (2008a), using a derivative devoid of LDH activity and overexpressing the native PCx gene (*pyc*), *C. glutamicum* R Δ*ldhA* pCRA717. In a first step, cells of this strain were grown under fully aerobic conditions. Then, the cells were harvested, washed and transferred to closed bottles, to give a high cell density of about 50 g_(DCW)_ l^−1^. With repeated intermittent addition of glucose and sodium bicarbonate, a final titre of 1.24 M succinate (146 g l^−1^) was obtained within 46 h, with a Y_P/S_ of 1.4 mol per mol of glucose (Okino *et al*., 2008a). The cells did not form any lactate; however, they produced significant amounts of acetate (0.3 M = 16 g l^−1^) as by-product.

**Figure 3:**
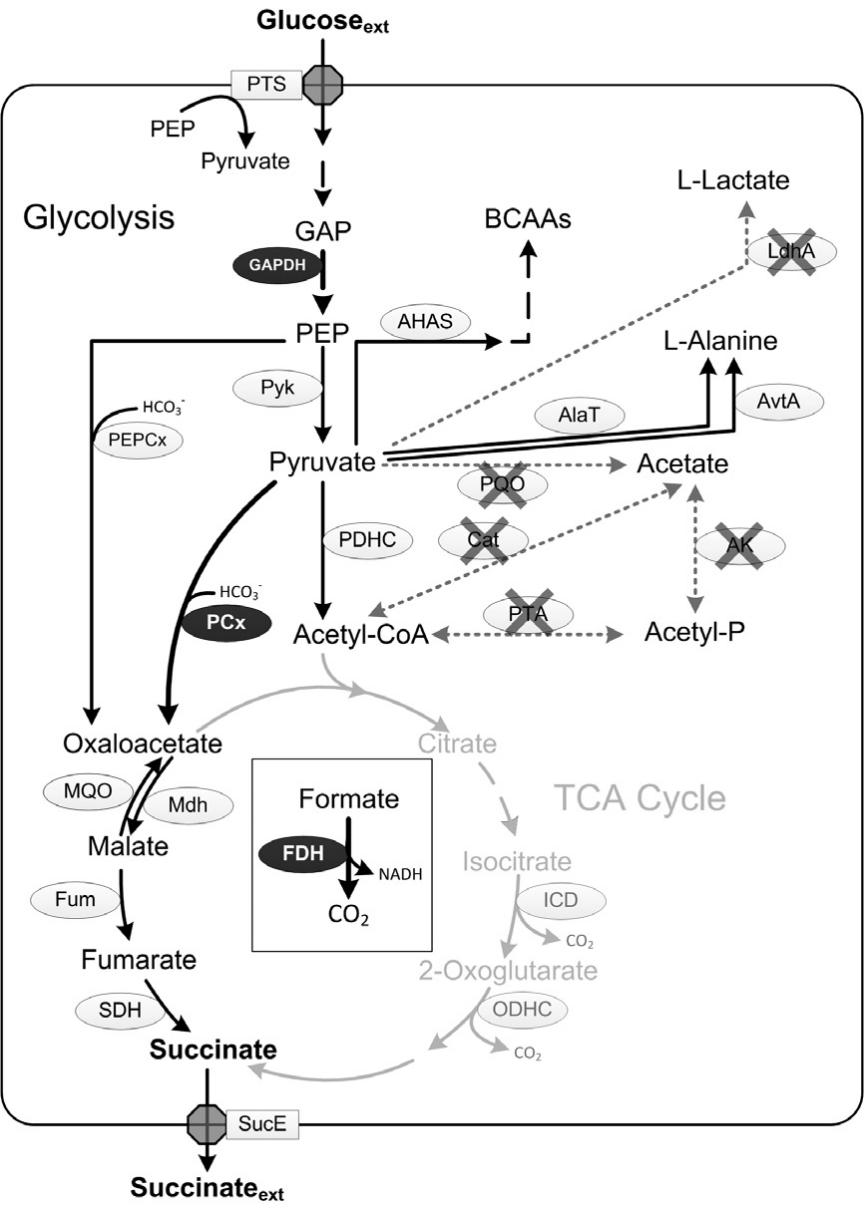
Schematic presentation of the central carbon metabolism of *C. glutamicum* BOL-3/pAN6-*gap* during anaerobic succinate production. For most abbreviations see legend to Fig. 1. Further abbreviations: BCAAs, branched-chain amino acids; LdhA, NAD^+^-dependent l-lactate dehydrogenase; SucE, succinate exporter; TCA, tricarboxylic acid. Dark ellipses indicate homologous/heterologous enzymes, crosses indicate inactivation of the enzyme by deletion of the respective gene. Dotted arrows indicate pathways not present due to gene inactivation, grey arrows indicate pathways not used in *C. glutamicum* BOL-3/pAN6-*gap*.

Recently, also Litsanov and colleagues (2012b) engineered *C. glutamicum* ATCC 13032 for high yield succinate production by further extending the experimental approach by Okino *et al*. (see above). Deletion of the LDH gene, chromosomal integration of an allele for a deregulated PCx (*pyc*^P458S^) and deletion of the genes encoding enzymes responsible for acetate synthesis (Δ*cat*, Δ*pqo*, Δ*pta-ack*) resulted in *C. glutamicum* BOL-2, that produces up to 116 mM succinate with a Y_P/S_ of 1.03 mol per mol of glucose, and pyruvate (23 mM) as well as 2-ketoglutarate (12 mM) as major by-products (Litsanov *et al*., 2012b). To increase NADH and CO_2_ availability and to increase the glycolytic flux, the authors then integrated the formate dehydrogenase gene *fdh* from *Mycobacterium vaccae* into the genome of *C. glutamicum* BOL-2 and additionally overexpressed the homologous glyceraldehyde-3-phosphate dehydrogenase (GAPDH) gene (*gapA*) from plasmid. In a fed-batch fermentation with glucose, formate and bicarbonate as substrates, the ultimate strain *C. glutamicum* BOL-3/pAN6-*gap* (see Fig. 3) produced 1.13 M succinate (134 g l^−1^) with a Y_P/S_ of 1.67 mol per mol of glucose (Litsanov *et al*., 2012b). Aside from succinate, 2-ketoglutarate (35 mM), malate (33 mM), acetate (20 mM), fumarate (13 mM), and pyruvate (6 mM) were formed as by-products.

**Figure 4:**
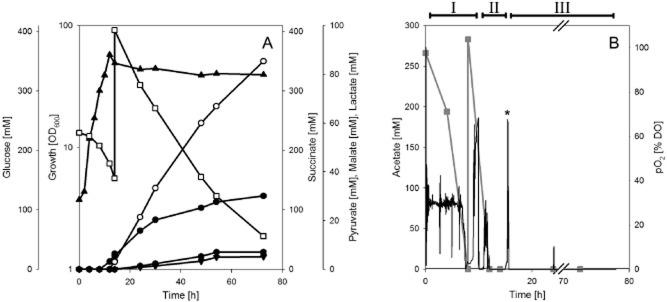
Growth, glucose consumption, and product formation (A) and acetate consumption and the course of the pO_2_ (B) during a representative pH-controlled tri-phasic fed-batch cultivation of *C. glutamicum* ELB-P in a 400 ml bioreactor with minimal medium, initially containing 4% (*w*/*v*) glucose, 1% (*w*/*v*) acetate and 6 mM l-alanine. (A) ▴, growth; □, glucose; •, pyruvate; ○, succinate; ▾, malate; 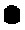, lactate. (B) Black line, pO_2_; grey line, acetate. Roman numerals indicate (I) aerobic growth phase, (II) self-induced microaerobic phase, and (III) oxygen deprivation by CO_2_ gassing. The pO_2_ peak at the end of the microaerobic phase (marked with the asterisk in 4B) indicated the end of aerobic growth, as O_2_ consumption stopped, leading to increasing DO in the medium. Immediately, aeration was replaced by CO_2_ sparging and the production phase started. A batch of glucose at the beginning of phase III should ensure carbon availability for succinate production. At least five independent fermentations were performed, showing comparable results.

In a further approach, the pyruvate-producing strain *C. glutamicum* ELB-P (see above and Fig. 2) was employed for succinate production (S. Wieschalka and B. J. Eikmanns, unpublished). Due to the inactivation of the PDHC, PQO, and LDH, this strain does not form significant amounts of acetate or lactate as by-products under any aerobic and anaerobic condition tested (Wieschalka *et al*., 2012). In contrast to the two-stage processes described above (i.e. aerobic growth in complex or minimal media and, after harvest of the cells and resuspension in new medium, transfer to sealed bottles or fermenters respectively; Okino *et al*., 2008a; Litsanov *et al*., 2012b), a one-stage fed-batch fermentation process with *C. glutamicum* ELB-P was established, combining biomass formation and succinate production in a single bioreactor. This process includes three phases: (i) an aerobic growth phase on glucose plus acetate, (ii) a self-induced microaerobic phase at the end of the exponential growth by minimal aeration, and (iii) an anaerobic production phase, realized by gassing the fermenter with CO_2_ (Fig. 4). This optimized process led to growth-decoupled succinate production of more than 330 mM (i.e. 39 g l^−1^) with a Y_P/S_ of 1.02 mol succinate per mol of glucose. The final Y_P/S_ obtained, together with the formation of pyruvate (about 30 mM) as by-product, however, still indicates a limitation, which might be overcome by increasing the carbon flux from PEP/pyruvate to OAA or by integration of the *M. vaccae fdh* gene and the use of formate as an additional substrate for reduction equivalents, as described above by Litsanov and colleagues (2012b).

The experimental setup of a one-stage process (consecutive aerobic growth and anaerobic production in a single bioreactor) as done with *C. glutamicum* ELB-P, see above) represents an industrially feasible process. However, a recent study on isobutanol production with *C. glutamicum* disclosed the differences in the production performance between two-stage fermentations (aerobic growth in complex or minimal media and anaerobic production in different containments, see above) and one-stage fermentations in a single bioreactor: The isobutanol Y_P/S_ in the one-stage fermentation was significantly lower (0.48 mol vs 0.77 mol of isobutanol per mol of glucose), indicating that the transition from the aerobic environment (growth phase) to the anaerobic environment (production phase) has a strong impact on the overall production behaviour (Blombach and Eikmanns, 2011; Blombach *et al*., 2011). Similarly, Martínez and colleagues (2010) recently observed that introducing a microaerobic phase at the end of the aerobic growth phase of an *E. coli* succinate-producer led to an adjustment of the enzymatic machinery and to improved succinate production under anaerobic conditions. To our knowledge, the physiological changes of *C. glutamicum* during a (slow or fast) shift from aerobic to anaerobic conditions have so far not been investigated. However, it can be foreseen that the insight into the metabolic adaptation of the cells to such alternating culture conditions will help to further optimize organic acid production by novel metabolic engineering approaches and also by applying optimally adapted process conditions.

The Y_P/S_ of the most efficient *E. coli* strains producing succinate under anaerobic conditions, *E. coli* SBS550MG/pHL413 and *E. coli* KJ134, were 1.60 mol and 1.53 mol succinate per mol of glucose respectively (Sánchez *et al*., 2005; Jantama *et al*., 2008; Table 1). Thus, both recombinant *E. coli* strains and in particular, *C. glutamicum* BOL-3/pAN6-*gap* (Table 1) showed higher Y_P/S_ than all known natural succinate-producing bacteria, such as *Anaerospirillium succiniproducens* (1.37 mol mol^−1^ of glucose; Glassner and Datta, 1992) or *Mannheimia succiniproducens* (1.16 mol mol^−1^ glucose; Lee *et al*., 2006). A further advantage of employing the recombinant *C. glutamicum* or *E. coli* strains is the potential use of mineral media, keeping production and purification costs lower than with *Mannheimia* or *Anaerospirillum*, which both require complex media. *C. glutamicum* BOL-3/pAN6-*gap* and *C. glutamicum* R Δ*ldhA* pCRA717 produced about threefold higher succinate titres than *E. coli* SBS550MG/pHL413 (Table 1) and thus, *Corynebacterium* seems to be the superior organism for succinate production.

Very recently, Litsanov and colleagues (2012a) reported also on aerobic succinate production with *C. glutamicum* for the first time. Deletion of the SDH genes initiated aerobic succinate production in *C. glutamicum* via glycolysis, PEP and/or pyruvate carboxylation, the oxidative branch of the TCA cycle, and the glyoxylate shunt. Acetate formation was mostly prohibited by shutdown of the known pathways for acetate synthesis, resulting in *C. glutamicum* BL-1 (genotype: Δ*sdhCAB*, Δ*cat*, Δ*pqo*, Δ*pta-ack*; Litsanov *et al*., 2012a). To reduce carbon-loss into cell mass, nitrogen-limited growth conditions were established, forcing the cells into a resting state after a certain period. With additional, plasmid-bound overproduction of both PEPCx and the PCx^P458S^-variant, final succinate titres and Y_P/S_ of up to 90 mM and 0.45 mol succinate per mol of glucose, respectively, were observed (Litsanov *et al*., 2012a; Table 1). Concerning the specific productivity of 1.6 mmol g_(CDW)_^−1^ h^−1^, *C. glutamicum* BL-1/pAN6-*pyc*^P458S^*ppc* showed the highest value described so far for aerobic succinate production from glucose with bacteria.

In comparison to other bacterial succinate producers, *C. glutamicum* BL-1/pAN6-*pyc*^P458S^*ppc* is exceedingly competitive in aerobic succinate production (Table 1). Lin and colleagues (2005) described various *E. coli* strains approaching the maximal theoretical Y_P/S_ of about 1 mol succinate per mol of glucose under aerobic conditions. *Corynebacterium glutamicum* BL-1/pAN6-*pyc*^P458S^*ppc* did not reach this high Y_P/S_, but the recombinant *C. glutamicum* strains produced significantly higher final succinate titres in minimal instead of complex media (Table 1).

## Production of organic acids with *C. glutamicum* from alternative substrates

Economical relevant and sustainable production of organic acids with microorganisms in an industrial scale is dependent on the use of low-cost carbon sources, in particular from renewable resources. So far, we focused in this review on the fermentative organic acid production from pretreated and purified carbon sources, such as glucose and glucose plus formate, since the most promising attempts to produce organic acids with *C. glutamicum* were made with these substrates. To simplify feedstock purchase and to improve the economic efficiency, utilization of alternative, crude materials is of great interest. However, *C. glutamicum* naturally cannot utilize certain industrially relevant substrates, such as glycerol, starch (from corn, wheat, rice, or potato), whey, straw, or hemi-and lignocellulose. Especially lignocellulose, consisting largely of cellulose, hemicellulose, and lignin, is a widely abundant and potentially attractive source of renewable feedstock. Hemicellulose, consisting mainly of glucose but also to a significant portion of C_5_ sugars (xylose and arabinose) (Wiselogel *et al*., 1996; Aristidou and Penttilä, 2000), can be depolymerized by chemical or enzymatic processes, and the resulting sugar mixtures are also of interest as alternative feedstock for *C. glutamicum*. Whereas some organisms (e.g. *E. coli*) are naturally able to consume the majority of sugars in the mixtures resulting from saccharification from hemicellulose, *C. glutamicum* needs metabolic engineering to expand the spectrum of sugars that can be utilized. Thus, the extension of the substrate spectrum of *C. glutamicum* to cheap, easily accessible and renewable monomeric and polymeric carbon sources is desired and therefore, an ongoing field of intensive research (Wendisch *et al*., 2006b; Blombach and Seibold, 2010; Rumbold *et al*., 2010; Okano *et al*., 2010; Becker and Wittmann, 2011).

Several attempts have been made to broaden the natural substrate spectrum of *C. glutamicum* towards starch (Seibold *et al*., 2006; Tateno *et al*., 2007), whey (Barret *et al*., 2004), rice straw and wheat bran hydrolysates (Gopinath *et al*., 2011), grass and corn silages (Neuner *et al*., 2012), glucosides and d-cellobiose (Kotrba *et al*., 2003), glycerol (Rittmann *et al*., 2008), amino sugars (Gruteser *et al*., 2012; Uhde *et al*., 2012) or pentose sugars for growth and for the production of amino acids or other value-added products (Blombach and Seibold, 2010; Jojima *et al*., 2010; Buschke *et al*., 2011; Schneider *et al*., 2011; Gopinath *et al*., 2012). The first approaches to extend the substrate spectrum especially for organic acid production were performed by Kawaguchi and colleagues (2006; 2008) and Sasaki and colleagues (2008; 2009). Plasmid-bound introduction of the xylose isomerase and xylulokinase genes (*xylA* and *xylB* respectively) from *E. coli* into *C. glutamicum* R enabled both aerobic growth on xylose as sole carbon source and production of l-lactate and succinate with resting cells under oxygen deprivation conditions (Kawaguchi *et al*., 2006). Although the sugar consumption rate and the specific productivity of the recombinant *C. glutamicum* CRX2 was lower with xylose than with glucose, the Y_P/S_ for succinate was even higher on xylose (0.42 mol mol^−1^) than on glucose (0.23 mol mol^−1^). In contrast, the Y_P/S_ for l-lactate was lower with xylose as substrate (1.06 and 1.36 mol mol^−1^ respectively; Kawaguchi *et al*., 2006). A similar behaviour was shown for succinate and l-lactate production from arabinose with *C. glutamicum* CRA1, which expresses the *E. coli* genes *araA*, *araB* and *araD* (encoding l-arabinose isomerase, l-ribulokinase, and l-ribulose-5-phosphate 4-epimerase respectively) and therefore is able to metabolize this C5 sugar (Kawaguchi *et al*., 2008). In this case, with 200 mM arabinose as substrate, the Y_P/S_ for succinate and l-lactate were 0.67 mol mol^−1^ and 0.75 mol mol^−1^ respectively (Kawaguchi *et al*., 2008).

Co-utilization of different C5 sugars with C6 sugars was investigated to study catabolite repression effects in *C. glutamicum* and to expand sugar utilization on conditioned hemi-and lignocellulosic biomass hydrolysates (Sasaki *et al*., 2008). These efforts resulted in a *C. glutamicum* strain harbouring *xylA* and *xylB* as well as *bglF^V317A^* and *bglA* (encoding PTS β-glucoside-specific enzyme IIBCA component and phospho-β-glucosidase respectively). This strain produced from a mixture of d-cellobiose (10 g l^−1^), glucose (40 g l^−1^), and d-xylose (20 g l^−1^) about 460 mM l-lactate, 110 mM succinate, and 30 mM acetate under anaerobic conditions, with a combined yield of 0.85 g acids per g of sugar (Sasaki *et al*., 2008). A combined strain, containing all named modifications for d-xylose, l-arabinose and d-cellobiose consumption, and additionally overexpressing the arabinose transporter gene *araE* from *C. glutamicum* ATCC31831, was even able to consume glucose (35 g l^−1^), d-xylose (17.5 g l^−1^), l-arabinose (7 g l^−1^), and cellobiose (7 g l^−1^) simultaneously and completely under oxygen-deprived conditions within 14 h (Sasaki *et al*., 2009).

Recently, Sasaki and colleagues (2011) developed a *C. glutamicum* strain overexpressing the mannose 6-phosphate isomerase and fructose permease genes *manA* and *ptsF* respectively. This strain consumed mannose and glucose simultaneously and produced about 400 mM l-lactate, 100 mM succinate and 30 mM acetate from a sugar mixture of 200 mM glucose and 100 mM mannose under oxygen deprivation conditions (Sasaki *et al*., 2011).

Litsanov and colleagues (2012c) very recently showed aerobic succinate production with glycerol as sole carbon source, by plasmid-bound transfer of the glycerol utilizing genes *glpFKD* from *E. coli* into *C. glutamicum* BL-1. Glycerol is a main by-product of biodiesel and bioethanol production (Yazdani and Gonzalez, 2007) and using this carbon source for the production of value-added chemicals (such as succinate), the economic efficiency of these biofuel production processes can be increased (Wendisch *et al*., 2011). Plasmid pVWEx1-*glpFKD* has previously been shown to enable growth and amino acid production of *C. glutamicum* on glycerol as sole carbon source (Rittmann *et al*., 2008). Consequently, using the conditions established for *C. glutamicum* BL-1/pAN6-*pyc*^P458S^*ppc* (see above), *C. glutamicum* BL-1 (pVWEx1-*glpFKD*) aerobically produced up to 79 mM succinate (9.3 g l^−1^) with a Y_P/S_ of 0.21 mol per mol of glycerol (Litsanov *et al*., 2012c). The specific succinate productivity of *C. glutamicum* BL-1 pVWEx1-*glpFKD* on glycerol was as high as for *C. glutamicum* BL-1/pAN6-*pyc*^P458S^*ppc* on glucose with 1.6 mmol g_(CDW)_^−1^ h^−1^. However, the volumetric productivity of 3.59 mM h^−1^ is the highest productivity so far described for aerobic succinate production (Litsanov *et al*., 2012c; Table 1).

In summary, the above mentioned studies showed the feasibility to expand the substrate spectrum of *C. glutamicum* to the main C5 and C6 sugars found in agricultural residues, in hydrolysed hemicellulose and lignocellulosic biomass, and to glycerol. For directed production of organic acids from hemicellulose feedstock, the modifications made for broadening the substrate spectrum and those made for optimal carbon flux to a desired organic acid must be combined. The successful aerobic production of succinate from glycerol instead of glucose by introduction of the glycerol utilizing genes from *E. coli* to *C. glutamicum* (Litsanov *et al*., 2012c; see above), is one such example and promises the feasibility of such approaches.

## Summary and outlook

Driven by old and new knowledge and genome-based metabolic and genetic engineering strategies, *C. glutamicum* has become a major candidate as platform organism for bio-based, industrial production of a variety of organic acids from renewable biomass. As outlined above and highlighted in Table 1, titres, Y_P/S_, and productivities of recently developed *C. glutamicum* producer strains are highly competitive, in several cases already superior in comparison to other bacterial, well-established production systems. From current studies on transcriptome, proteome, metabolome, and intracellular fluxes (Vertes *et al*., 2012), from recent advances in evolutionary engineering tools (Becker and Wittmann, 2011), and from recent development of plasmid addiction systems (Schneider *et al*., 2012) and of single cell approaches (Binder *et al*., 2012; Mustafi *et al*., 2012), it can be expected that a variety of further metabolic (or genetic) targets for strain development/improvement will be identified in *C. glutamicum*. Future approaches to optimize organic acid production certainly will not only aim at substrate flexibility (low cost and eco-efficient feedstocks), product extension (e.g. fumarate, malate, or itaconate), and/or the paths from a substrate or substrate mixtures to the desired products (i.e. substrate uptake, central metabolism, precursor supply, synthetic pathways, and export of the respective organic acid). They will also focus on maintenance of a well-balanced redox state within the cells, on optimal adaptation of the cells to alternating culture conditions (e.g. shift from aerobic to anaerobic conditions), on strain robustness, and on an increased acid-resistance of the producer strains. Tolerance to organic acid stress represents a highly relevant factor for process design and downstream processing of large-scale production processes, since organic acid recovery from low pH fermentation broth in general is more cost-efficient than from neutral broth. However, the achievements obtained in the last 6 years and the wealth of new knowledge about the physiology, the metabolism and its regulation, and the proven production capabilities of *C. glutamicum* bode well for the implementation of this organism as a platform for new and even more (cost-)efficient processes for the production of a variety of organic acids and also of other specialty, fine and bulk chemicals.

## Conflict of interest

None declared.
